# From grid cells and visual place cells to multimodal place cell: a new robotic architecture

**DOI:** 10.3389/fnbot.2015.00001

**Published:** 2015-04-07

**Authors:** Adrien Jauffret, Nicolas Cuperlier, Philippe Gaussier

**Affiliations:** ETIS, UMR 8051/ENSEA, Université Cergy-Pontoise, CNRSCergy, France

**Keywords:** neural network, entorhinal cortex modeling, grid cells, place cells, mobile robot

## Abstract

In the present study, a new architecture for the generation of grid cells (GC) was implemented on a real robot. In order to test this model a simple place cell (PC) model merging visual PC activity and GC was developed. GC were first built from a simple “several to one” projection (similar to a modulo operation) performed on a neural field coding for path integration (PI). Robotics experiments raised several practical and theoretical issues. To limit the important angular drift of PI, head direction information was introduced in addition to the robot proprioceptive signal coming from the wheel rotation. Next, a simple associative learning between visual place cells and the neural field coding for the PI has been used to recalibrate the PI and to limit its drift. Finally, the parameters controlling the shape of the PC built from the GC have been studied. Increasing the number of GC obviously improves the shape of the resulting place field. Yet, other parameters such as the discretization factor of PI or the lateral interactions between GC can have an important impact on the place field quality and avoid the need of a very large number of GC. In conclusion, our results show our GC model based on the compression of PI is congruent with neurobiological studies made on rodent. GC firing patterns can be the result of a modulo transformation of PI information. We argue that such a transformation may be a general property of the connectivity from the cortex to the entorhinal cortex. Our model predicts that the effect of similar transformations on other kinds of sensory information (visual, tactile, auditory, etc…) in the entorhinal cortex should be observed. Consequently, a given EC cell should react to non-contiguous input configurations in non-spatial conditions according to the projection from its different inputs.

## 1. Introduction

In robotics, getting a robust localization is crucial to achieve navigational tasks. Indeed, Simultaneous Localization and Mapping (SLAM) is a problem that has concentrated much of the research effort in autonomous robot navigation for more than 20 years (Chatila and Laumond, 1985; Durrant-Whyte and Bailey, 2006). The oldest approach proposed to solve SLAM is the probabilistic one, based on the Extended kalman Filter (EKF) (McElhoe, 1966). The complexity of EKF is quadratic with respect to the landmark's number and thus it behaves badly in large environment. This algorithm is also very sensitive to bad associations. To cope with these limitations, numerous methods have been proposed. One can refer to Thrun et al. (2005) and Thrun (2008) for a review.

Taking inspiration from nature, bio-inspired robotics proposes robotic control architectures based on models of animal's spatial cognition. The goal of this approach is both to better understand the cognitive processes underlying animals behaviors and to endow robot architectures with robust and adaptive behaviors. Neuro-Ethological studies of mammals performing navigation tasks show that a wide variety of sensory modalities can be combined and processed to yield position information (Etienne and Jeffery, 2004). The discovery of place cells (PCs) in the rat hippocampus (HS) (O'Keefe and Nadel, 1978) has emphasized the encoding of a neural representation of allocentric space used by mammals. These cells exhibit place related activities (place field) and are thought to be used to navigate. Other neurons found in mammal brain seem to act as an internal compass. These cells fire in relation to the animals directional heading and are named Head direction cells (HD cells). HD cells have been first identified in the post-subiculum (Taube et al., 1990) and then they have been found in several structures like in the anterior thalamic nuclei (Taube, 1995) and in the retrosplenial cortex (Chen et al., 1994). For a short and recent review on HD cell firing properties and the neuronal structures where they have been found, one can refer to Winter and Taube (2014). More recently, the entorhinal cortex (EC) has been the focus of attention since the striking discovering of grid cells (GC) in the dorso-lateral band of the medial EC (dMEC) (Hafting et al., 2005). When recorded in sufficiently large environments, these cells present spatial firing fields forming a regular hexagonal pattern or a grid that tiles the environment explored by the rat. HD cells and conjunctive GC have also be reported in dMEC (Sargolini et al., 2006). In rodents, GC activities are anchored to external landmarks. But grids also persist in their absence. In dark condition, the average firing rate and spacing of GC seem unchanged but a decrease in the spatial correlation of the rate maps underlines a dispersal or displacement of the vertices (Hafting et al., 2005).

Numerous models of GC have been proposed since 2005. They mainly differ by the way they code positional information, how this information is updated when the animal moves and how the read out of this code is performed (Zilli, 2012). A first class of models encodes positional information as phase difference between oscillators and updates it via frequency modulation (Burgess et al., 2007). Read-out mechanism in these models is commonly based on temporal interference between the oscillators. A second class uses an attractor network (i.e., continuous attractor network) to code spatial information (Fuhs and Touretzky, 2006; McNaughton et al., 2006), where the attractor is shifted in the network according to HD input signal. Most of these models use a direct read-out mechanism (location of the activity bubble). Finally a few models use the firing rate of single cells as coordinate where the read-out is performed as a spatial interference mechanism (Gaussier et al., 2007; Hasselmo and Brandon, 2008). These two models differ by the way they update the network : it is based on a firing rate modulation in Gaussier et al. (2007) and based on a frequency modulation in Hasselmo and Brandon (2008). Recently *hybrid models* relying both on a continuous attractor network for positional information encoding and on the interference mechanism to read-out have been proposed (Welday et al., 2011; Mhatre et al., 2012). Despite a huge amount of theoretical models, little is known about the requirements needed to replicate GC activities in real robotic experiments, how these models behave with real world *noisy* data. Indeed, most computational models of biological neuronal network are often tested using world models which have little resemblance to natural stimuli (movements in a discrete space, use of a uniform noise in a continuous environment, alignment of robot movement with the grid directions, recalibration with *ad-hoc* stimuli). Only a very few of these works were tested on robotic platform (Milford et al., 2010). Using robots allow to test how brain models react to environmental constraints close to those the animals have to face (for instance how to keep coherent and precise grid-like properties?). In this paper, our robot is used as a tool to study in “real world” conditions the coherence and the dynamics of HD cell, GC, and PC models in a simple yet real navigation task and to address the following questions: What are the constraints implied by a bio-inspired model closing the sensory-motor loop? At a behavioral level, does the generalization capability of the resulting place recognition allows learning an homing behavior or a route as a sensory-motor attraction basin?

We present in this paper a robotic implementation of a model exhibiting GC firing patterns. This model is based on a residue number system (Gaussier et al., 2007). Unlike most GC models, we propose that GC are not processing path integration (PI) but take this information as input instead. Indeed, several models explain how animals can compute PI (Hartmann and Wehner, 1995; Wittmann and Schwegler, 1995; Arleo and Gerstner, 2000). There are also evidences for the involvement of parietal cortices in PI (Parron and Save, 2004). In our model, as in Wittmann and Schwegler (1995), long-term path integration is performed over a one dimensional neural field. This kind of representation is well-suited to sustain homing behavior as it gives a direct access to the homing vector. We argue in this paper that the spatial grid pattern of GC activities can arise from a compression of this PI information.

Our experimental results on robots underline the key role played by visual inputs to maintain GC firing pattern over long periods. Without visual cues, GC firing activity does not correspond to a grid pattern but seems scrambled. A simple mechanism exploiting visual information can be used to recalibrate path integration in order to keep cumulative errors sufficiently low to obtain the typical GC firing pattern. Several experiments to study the impact of the model parameters and the effect of the different error sources over the grid cell pattern have been performed.

PC can be easily generated from GC (Gaussier et al., 2007), using a simple competitive learning combining the activities of several GC. This paper shows that a few parameters can control the size of the generated place field and thus control the generalization capability of place recognition. To test on a real robot the interest of the PCs obtained from GCs a simple fusion model has been used to combine them with visual place cells (using a simple conditional association mechanism). These visual place cells look like the large and noisy cells observed in the ventral medial entorhinal cortex (vMEC) (Quirk et al., 1992) and could play a key role in hippocampal cells activity (Poucet et al., 2014). Combining multimodal place cells from both visual place cell and GC inputs brings more robustness for the navigation in difficult environments as already shown by behavioral studies on animals and robot localization works (Filliat, 2003). We simultaneously recorded the activities of visual place cells (VPC), place cells generated from grid cells (PredVPC) and multimodal place cells (MPC) to analyze and compare the contribution of these different sources of information. Finally, experiments, we show how this simple model of MPC behaves when a conflict occurs between both inputs (when the robot is kidnapped and passively moved to a new location).

In following sections, we first describe the different parts of our model and how they interact. We then present the results of experiments performed to analyze the effects of the model parameters and to study the interactions between the different neural networks involved in our model. Finally, this paper ends with a discussion and some predictions related to the fact the GC would not be the result of a specific system devoted to navigation but the result of a general compression property from the associative cortical areas to the hippocampal system.

## 2. Materials and methods

In this section, the different neural networks necessary to compute robust GC are described. Three networks developed in previous works will be introduced: a model of VPC, a model of HD cells (that act as an internal compass) and a neuronal network relying on this internal compass to perform PI. A mechanism, based on associative learning between VPC and PI cells, will be used to limit the path integration error. Next, our GC model based on a compression of the path integration (PI) activity will be presented. Finally, a simple model of MPC merging GC and VPC activities will be proposed in order to evaluate the GC model in real life experiments. To simplify the simulations, in all our neural networks, the firing rate of the neurons will be represented by a normalized firing rate ranging from 0 to 1 (maximal frequency of activation).

### 2.1. Modeling place cells from visual information

Among other things, vision (as an exteroceptive modality) allows the animal to localize himself by the extraction of distant landmarks. Different models of biological vision-based navigation use the azimuth of the landmarks (Cartwright and Collett, 1983) or the conjunction of landmark identifications and their azimuths (Gaussier and Zrehen, 1995). A place can be recognized using only visual information as a configuration of landmarks and their angular position (either relative or absolute). In the following, we will refer to these cells as Visual Place Cells (VPCs). VPC could result from a biologically plausible model of the hippocampus we developed in previous works (for more details and equations one can refer to Gaussier et al., 2002; Banquet et al., 2005; Cuperlier et al., 2007). The VPC could be locate in the superficial layer of the ventral medial entorhinal cortex which receive inputs from the visual association cortex (through the perirhinal and post-rhinal cortices Agster and Burwell, 2013).

**Figure 1:**
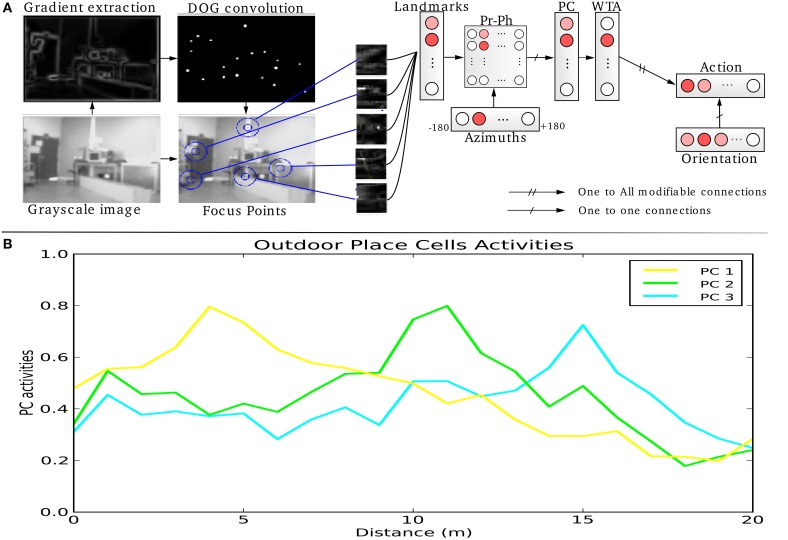
**(A)** Visual Place Cells model: A pan camera picks up 15 images (320 × 240 pixels) over a 360° panorama. Each gradient image is convolved with a difference of gaussian filter (DoG). Local maxima of the resulting image correspond to points of interest on which the system focuses on to extract local views and their corresponding azimuths. A Visual Place Cell (VPC) learns to recognize a specific landmark-azimuth constellation from the merging of perirhinal and parahippocampal structures (Pr-Ph). An action is associated with this PC. This association is learned by a least mean square algorithm. Then, the system automatically moves in the learned direction when the associated PC wins. **(B)** Activity of 3 visual place cells recorded on a linear track in a real outdoor environment. The different maxima of activity correspond to the learned positions of the associated cells. Our architecture provides good generalization properties since activities present large place fields.

In our VPC model, a panoramic image is analyzed sequentially in order to extract salient points (landmarks) in the scene. An embedded pan-tilt camera allows the capture of multiple images corresponding to a 360° panorama (15 images per panorama). A low resolution gradient extraction convolved with a difference of gaussian filter (σ_1_ and σ_2_ parameters) allows to highlight a set of salient points (curvature points) in the scene (see Figure 1A). An internal attentional mechanism is used to focus on each salient point. In our model, landmarks are made from local views corresponding to a small circular image centered on each focus point. A log polar transform mimicking the projection of the retina onto the primary cortical areas is used to improve the pattern recognition against small image rotations and scale variations. The neural network learns and recognizes a place as a constellation of landmarks and their azimuths in the visual scene. In the simplest versions the azimuth is computed from a magnetic compass but can be replaced by a visual compass (Delarboulas et al., 2014) or even be replaced by the relative angle between the considered landmark and another landmark (Gaussier et al., 2002).

Local views correspond to “what” information coded in the perirhinal cortex or in other areas of the ventral visual pathway of the rat temporal cortex (Kolb and Tees, 1990). The azimuths of these local views (“where” information) are provided by the parietal cortex through the parahippocampal region. Azimuths are coded on a neural field using a diffusion mechanism centered on the preferred direction, providing generalization capabilities (Georgopoulos, 1988). The merging of “what” and “where” information may be performed in the superficial layer of the entorhinal cortex or in the post-rhinal cortex (Suzuki et al., 1997; Burwell and Hafeman, 2003).

This model of VPC is able to categorize and to recognize different places in the environment. Activities of the different place cells depend on the recognition level of the landmarks. Robustness comes from the large number of local views extracted (75) and the only use of a competition mechanism between place cells: only the rank in the competition matters. The absolute value of VPC activity is not significant by itself while it can be high or low overall, depending on visual changes that appear in the environment.

Activity of VPC shows a peak for the learned locations, even in outdoor conditions (see Figure 1B). The PC generalize quite correctly over large distances (2–3 m indoor and 20–30 m outdoor).

### 2.2. Modeling head direction cells and path integration mechanism

In contrast with most models, we propose that the GC activity results from the projection and merging on the dMEC neurons of extra hippocampal PI activity.

**Figure 2:**
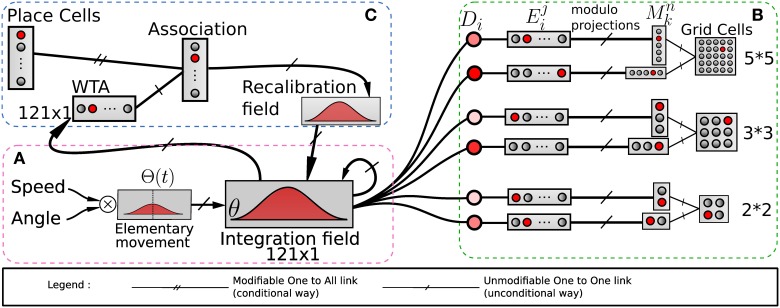
**Model of grid cells from path integration**. **(A)** Linear speed and absolute orientation can be used to characterize movement unit and so generate global path integration on a neural field. **(B)** Path integration neural field is used to build grid cell activities without any Cartesian map. Activities of randomly chosen pairs of neurons *D_i_* in the field θ are discretized on other fields *E*. Those fields are compressed by simple modulo projections on other fields *M*. The conjunction of 2 codes of 2 projections is sufficient to obtain grid cells. **(C)** A recalibration mechanism allows the system to limit a cumulative error on this field. This mechanism learns to associate the maximum activity (WTA) of the path integration field with the most active visual place cell (VPC) via a least mean square learning algorithm. It allows the system to recalibrate itself when it later recognizes known visual place cells.

Our PI model is directly inspired from Wittmann and Schwegler (1995) and Etienne (1998) (see Figure 2A) and it makes the hypothesis that HD cells provide the directional heading components to the path integrator (see also the model of Kubie and Fenton (2009) using the same assumption). This is consistent with several studies (Frohardt et al., 2006; Valerio and Taube, 2012) showing HD cells seems to be critical for navigation tasks based of PI. The HD cells are supposed to act as an internal compass since these cells discharge as a function of the direction in which the animal's head points in the horizontal plane of the environment, independent of the animal location. But heading direction are not sensitive to Earth's geomagnetic field, they are rather dependent on landmarks (vision) and self-motion cues (vestibular and proprioception). In Delarboulas et al. (2014) a detailed model of HD cells for robotics experiments is proposed. This model uses a dynamic neural field to merge both allothetic (a visual compass and/or a magnetic compass) and proprioceptive (robot odometry) information in order to compute the current heading of the robot.

In the present paper, path integration (PI) is performed on a discretized neural field of *N* neurons from the temporal integration of information related to small robot displacements (see Gaussier et al., 2007). We suppose that the neural field (*D*) for PI has the same topology and size (*N* neurons) than the input field (*V*) coding for the direction of the current movement. This *V* field takes as input the HD cells activities and codes it such as its activity has a non-negative cosine shape. At each time step (*t_s_*), the heading of the robot is coded on a circular and uniform neural map (*V*) covering 360°.

(1) $$V_{i}(\phi (t_{s}))\;=\;1+cos(\phi (t_{s})-\theta_{i})$$

(2) $$\theta_{i}\;=\;-2\pi \frac{i}{N}$$

The maximum of activity on V is centered on the direction of the current movement (ϕ(*t_s_*)). Activity of the neurons (*D_i_*) of the field performing the PI is simply computed as the sum of its previous activity (by mean of recurrent connections) with the corresponding activity of neuron *V_i_*:

(3) $$D_{i}(t_{s})={\left[D_{i}(t_{s}-1)+\alpha V_{i}(\phi (t_{s}))\right]}^{+}$$

(4) $${\left[x\right]}^{+}\;=\{\begin{array}{ll} x\; & \;if\;x\;\;>\;\;0\\ 0 & otherwise. \end{array}$$

α is a gain factor used to scale the length of the current movement on the neural field (0 < α < 1). Neurons in the field *D* have a maximum of activity for the direction of the global movement. The activity level of this neuron is proportional to the distance traveled from the starting point (where the last reset occurs). Proof and examples of this PI mechanism can be found in Gaussier et al. (2007). Plausibility of this model, the use of a cosine function (that can be replaced by a gaussian function or any other function having a bell curve activity), as well as the problem of the saturation of the field were discussed in Gaussier et al. (2007). The present paper does not intent to discuss further these issues. We simply make the assumption that a PI mechanism can compute a global path vector on a population of neurons and that it can serve as main input of our GC model.

When tested on a robot, PI errors mainly occur when the robot is turning (angular errors coming from the HD cell activities). To limit this issue, displacement distance is obtained directly from the robot odometry (for distance information) while a magnetic compass is used to obtain the orientation information since odometric information alone was too noisy on our robot to obtain an usable path integration. Relying on this multimodal path integration allows limiting the angular error on the robot odometry. Note that, even when considering the ideal case of no noise on the distance and angle measures, previous simulation results show that the model used for PI introduces errors due to the discretization of the angles (size of the neural fields). These errors quickly degrade regular pattern activities by spreading over neighboring areas so much that the global activity appears randomly distributed over space (Gaussier et al., 2007).

A solution consists in recalibrating the PI at well-known locations thanks to visual cues or any salient information in the environment (Etienne and Jeffery, 2004). Thus, in our previous model (Gaussier et al., 2007) a reset of the PI field can be performed when a binary signal *r* is set to one. This *r* signal can be triggered when a given VPC exhibits a strong activation. In this paper, we added the possibility to force the value of neuron activities in the PI field to a previously learned state associated with the current winning VPC (see Figure 2C). In our model, each time the robot recruits a new VPC (learns to recognize a new place) it associates this cell with the current PI activity. Later, the recognition of the same place allows to set the PI activity to the previously learned values and thus limits cumulative errors. The proposed mechanism is quite similar to the one proposed by Strosslin et al. (2005). It mainly differs by the way PI PI is taken into account. Our model relies on a normalized least mean square algorithm (NLMS) (Haykin, 2002) and is tested on a real robot. It is important to note that a reset occurs on PI field each time the first learned VPC wins the competition since this cell has been associated with the initial state of the PI field (set to zero). This place can be defined as a goal place to reach in a homing behavior.

PI recalibration occurs when a place is well-enough recognized. This leads to the definition of level of confidence in place cells recognition. To be efficient, the recalibration zone has to be narrow. In our experiments on GC, we choose to trigger a recalibration signal according to thresholds on VPC activity. A recalibration happens when the recognition level of the winning VPC satisfies 2 conditions: its activity must be over a first threshold (absolute threshold) and the difference in activity with the second most activated VPC must be over a second threshold (relative threshold). These 2 conditions allow avoiding recalibration in ambiguous places. Threshold values can be found in the Appendix in Supplementary Material. An even narrower recalibration zone can be easily obtained by using an other categorization input (like the recognition of ultrasound sensors profile at the goal place) in conjunction of the VPC recognition.

### 2.3. Modeling grid cells from extra hippocampal path integration

Our model of GC is based on various modulo's operators applied on PI (see Figure 2B). The activity *D_i_*(*t*) of a neuron belonging to the PI field (associated with direction θ_*i*_) is discretized over a new field of neurons:

(5) $$E_{j}^{i}(t_{s})=\{\begin{array}{ll} 1\; & \;if\;j\;=\;floor(\frac{D_{i}(t_{s}).N_{E}}{D_{max}})\\ 0\; & \;otherwise. \end{array}$$

Where *D_max_* is the maximum value of the distance that can be computed by the neural field and *N_E_* is the number of neurons on each field used to discretize the analog activity on the PI field. *i* is the index of the neuron from which is read the *D* field to generate the corresponding *E* field. Then a modulo operator is used to compress the field *E^i^* by projection in a field (*M^n^*) of smaller size:

(6) $$M_{k}^{n}(t_{s})=\{\begin{array}{ll} 1\; & \;if\;k\;=\;\arg\max_{j}(E_{j}(t_{s}))\;mod\;MG^{n}\\ 0\; & \;otherwise. \end{array}$$

with *MG^n^* the value of the modulo used to build the grid *n*. Values of the *MG^n^* factors represent a distance in an abstract unit corresponding to $\frac{D_{max}}{N_{E}}$. The sizes of *M* fields used in our experiments are given in annexe. The conjunction of 2 of these modulo projections (*M^n^* fields in Figure 2B) is sufficient to obtain a set of GC. Gaussier et al. (2007) have shown that learning these conjunctions is equivalent to learn a “AND” configuration since only two inputs are active. In our robotic model we thus used this simplified equation (without learning):

(7) $$G^{n}(t_{s})\;=\;M_{1}^{n}(t_{s})⊗M_{2}^{n}(t_{s})$$

with *M^n^*_1_ one of the two fields (belonging to a given field *E^i^*) and *M^n^*_2_ the other one. *G^n^_l_* is the activity of the *lth* GC neuron in the layer corresponding to modulo *n*. The spacing of the grid is determined by the modulo value *MG^n^* and the radius of the firing field by the discretization factor of PI neurons.

The absolute difference between θ_1_ (corresponding to a given neuron in *D*) and θ_2_ (corresponding to the other neuron chosen in *D*) determines the grid orientation. The compression factor of the modulo projection (corresponding to the ratio between input and output group) determines the grid spacing. Each neuron on the output layer of a given modulo generates a grid of the same orientation and spacing but with different phases. Figure 2B shows an example with three layers of GC corresponding to three different modulo values.

### 2.4. Building robust multimodal place cells from visual cells and grid Cells: a conditional association

In Gaussier et al. (2007), authors have proposed to generate PC from GC as the result of a pattern recognition process of the grid activity. A competitive learning mechanism (an online Winner Takes All in our work) similarly to the one proposed in Rolls et al. (2006) is used. One surprising result is that the generated place fields are very narrow as compared to the VPC obtained from the landmark ^*^ azimuth constellation while using the same competitive learning mechanism. In the model presented in Section 2.2, all grids present binary fields (activated or not) so that the pattern generated by the conjunction of 3 grids is a three-steps stair shaped (see result in **Figure 4**). We provide simulation Experiments (6, 7) that extend our previous works by studying two key parameters controlling the generated place fields. Experimental results in Section 2.5.4 show that place field of PC can be controlled by these two parameters but larger GC networks (more than 1000 neurons) are required. To overcome this limitation and keep a number of simulated GC neurons that is still compatible with real-time requirement of the robot control architecture, we chose to keep only three different networks of GC with binary output. To obtain PC with a place field size adapted to robot navigation, local lateral connections on each map of GC were added in order to allow some activity. These connections have positive weights decreasing as a function of the distance to the departure neuron. These new inputs allow our GC to provide analog responses (ϵ [0;1]) instead of binary outputs. This diffusion mechanism can be seen as the result of a convolution of the binary output of each GC with a gaussian mask. They allow to activate more than one neuron in each grid layer and thus give to the system more generalization capabilities (see Figure 4). The model described in the following is relying on GC with non-binary activity.

As indicated previously, hippocampal place cells (or place cells in the dentate gyrus) are also driven by visual inputs. One solution is then to combine the VPC, presented in Section 2.2 (driven only by visual inputs) and the new neural mechanism exploiting PI to model GC. In this section, we propose a simple merging mechanism that benefits of both modalities and is particularly useful when vision leads to an ambiguous recognition.

**Figure 3:**
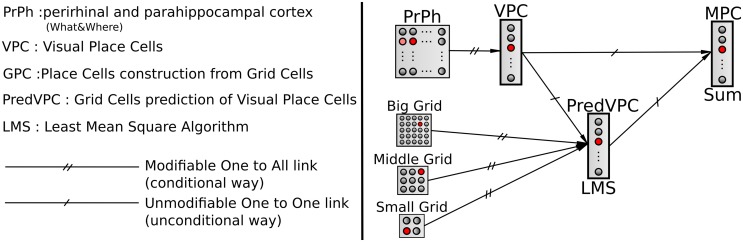
**A conditional association to merge visual and grid information**. Associations between visual place cells (VPCs) as unconditional stimulus and place cells predicted by grid cells (PredVPCs) taken as conditional stimuli.

We choose to model hippocampal PC by learning associations between VPC states and GC states. Associations are performed by a normalized least mean square algorithm (NLMS) that tries to predict the visual state (unconditional stimulus) from GC activity (conditional stimulus). Finally, a simple weighted sum is used to merge the activity of the VPC with the predicted activity from the GC (see Figure 3). This sum allows studying the contribution of one modality in the MPC response ^1^. The activity of a multimodal place cell *MPC_m_* is given by:

(8) $$MPC_{m}(t_{s})=\eta \;VPC_{m}(t_{s})+(1-\eta )PredVPC_{m}(t_{s})$$

with *VPC_m_* the activity of the corresponding VPC, *PredVPC_m_* the activity of the corresponding place cell predicted by the grids (NLMS output), η ϵ [0;1] a weighting factor and *m* ϵ [0;M] the index of the cell. We fixed in this paper the weighting factor η equals to 0.5 giving thus both sources the same weight.

**Figure 4:**
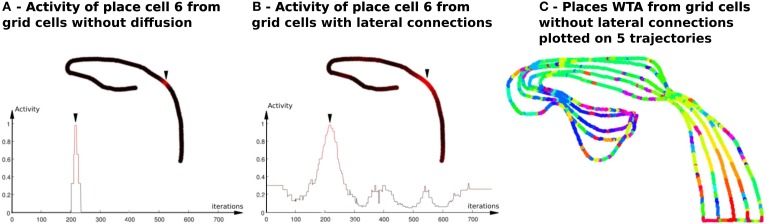
**Activity of place cells predicted by grid cells (model relying on a WTA)—Impact of the diffusion mechanism on GC networks (Experiment 6). (A)** PredVPC from binary grid cells (without lateral connections). Top : Activity of the 6th PredVPC is recorded in space along a multi-room path (*path*1 described in Section 2.5.2). A red color means a high activity and a black one indicates a low activity. Location of the cell numbered 6 in the path is marked by an arrow. Bottom: Activity of the 6th PredVPC is recorded in time. On the x axis, iterations are proportional to the distance the robot has traveled (constant speed). The place field is a thin three-steps stair shaped. **(B)** PredVPC from grid cells with lateral connections (positive weights that decrease as a function of the distance with neighboring neurons). Top : Activity of the 6th PredVPC is recorded in space along the same path than in panel **(A)**. A red color means a high activity and a black one indicates a low activity. Location of the cell numbered 6 in the path is marked by an arrow. These connections allow to spread activity on neighboring grid cells. Bottom: Activity of the 6th PredVPC is recorded in time. On the x axis, iterations are proportional to the distance the robot has traveled (constant speed). PredVPCs show larger place fields allowing generalization capabilities. **(C)** Multiple place fields with PredVPCs from the binary grid cells (no diffusion). This figure shows the identity of the highest PredVPC (resulting from a Winner Takes All competition mechanism) superimposed to the path. At each time step a colored dot is plotted. Each color identifies the PredVPC with the highest response at the given place. It exhibits a lot of recognition errors (same color for different locations) that can be easily removed by adding the diffusion mechanism (see **Figure 11**).

Recently, it has been highlighted that even if the medial entorhinal cortex (with its GC) and the ventral medial entorhinal cortex are both involved in hippocampal place cell activities, they could play a different role according to ambient light conditions (luminous or dark environments) (Poucet et al., 2014). The impact on MPC activity of the parameter η has to be done in a further study.

The activity of a place cell predicted by GC is given by:

(9) $$PredVPC_{m}(t_{s})=\sum_{l=0}^{L-1}w_{l}(t_{s})G_{l}(t_{s})$$

Where *w_l_* is the weight of the synapse coming from the corresponding GC *G_l_*.

Evolution of this weight is given by the normalized least-mean-square learning rule:

(10) $$\begin{array}{l} w_{l}(t_{s})=\\ \{\begin{array}{ll} 0\; & \;if\;t_{s}=0\\ w_{l}(t_{s}-1)+\frac{\begin{array}{l} \lambda_{m}(VPC_{m}(t_{s}-1)-\\ PredVPC_{m}(t_{s}-1))G_{l}(t_{s}-1) \end{array}}{\sum_{l\;=\;0}^{L-1}G_{l}{\left(t_{s}-1\right)}^{2}}\; & otherwise\;(t_{s}>0). \end{array} \end{array}$$

(11) $$\lambda_{m}=\lambda .\gamma .\beta_{m}$$

With λ_*m*_ a local learn factor depending on a constant λ ϵ [0;1], a binary neuromodulation factor γ (0 or 1, controlled by human) and a binary winner-takes-all β_*m*_ so that:

(12) $$\beta_{m}=\{\begin{array}{ll} 1\; & \;if\;m\;=m^{\prime }\;with\;m^{\prime }\;=\;argmax_{m}(VPC_{m})\\ 0 & otherwise. \end{array}$$

### 2.5. Experimental setup and description of the different experiments

#### 2.5.1. Robotic platform

**Figure 5:**
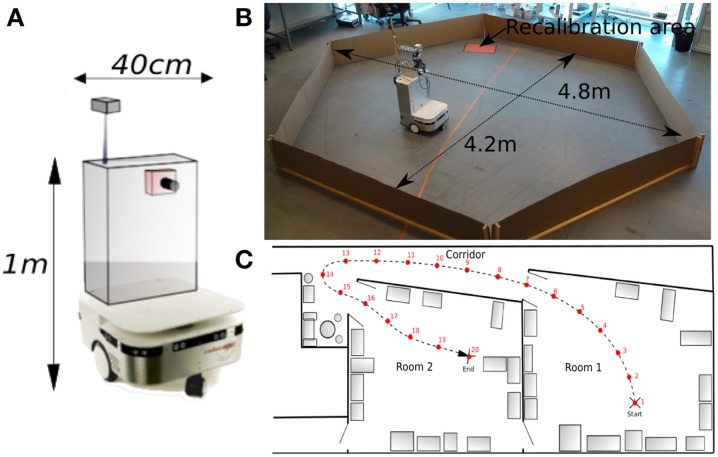
**(A)** Robotic platform used to perform our experiments. **(B)** Grid cells Experiments (1–5): Our 1 m high, 40 cm wide robot is freely moving in a 4 m diameter hexagonal enclosure using random movements and obstacles avoidance. The system learns by a few place-action associations how to return to a “home” location (marked by a red paper on the floor), that allows the robot to recalibrate itself. An internal time counter pushes the robot to go recalibrate itself every X minutes (X depending on the experiment), in order to limit cumulative errors of path integration. **(C)** Multiroom environment used to study place cells response (Experiments 8–11). The robot learns 19 regularly-spaced places (each 1.5 m), starting from a room, passing through a corridor and ending on a second room.

All robotic Experiments (1–5, 8–11) presented in this paper were performed on a Robulab 10 from Robosoft. It is a 40 cm large and 1 m tall robot (see Figure 5A). 10 ultrasound sensors (6 in front and 4 at the back of the robot) detect obstacles at 40 cm distance. These sensors are only used for obstacle avoidance (at the exception of the Experiments 3b, 5). The robot is equipped with 2 driving wheels centered on each side and 2 free wheels (one in front and one at the back of the robot) so that it can turn on itself. Robot displacements are controlled by sending a linear speed and a rotational velocity to the robot hardware controller (see Experiment Parameters in Appendix for values)(see Figure 5).

A camera with a field of view of 90° (fish-eye lens) and a resolution reduced to 320 × 240 pixels is mounted on top of the robot. A pan mechanism allows the camera to take 15 images covering a 360° visual panorama of the surrounding environment. A Difference Of Gaussian (DoG) filter is applied on each image to highlight salient points in the scene. Overlaps between successive images allow avoiding side effects. The system picks up 5 landmarks per image, representing a total of 15 × 5 = 75 landmarks for each panorama.

A magnetic compass is also used to get an absolute angular reference and is used as an input of our HD cell model. Its accuracy is around 1°. As seen before, a visual compass (Giovannangeli and Gaussier, 2007; Delarboulas et al., 2014), and an inertial sensor could replace it for a more biologically plausible solution (but it would make the analysis of the results more difficult).

Our control architecture relies on a real time neural networks simulator named Promethe (Matthieu et al., 2008) running on an Intel I5 (2.40 Ghz) CPU embedded in the robot. Working stations are only used for remote control and debugging purpose.

#### 2.5.2. Robot environment

The first Experiments (1–5) aim at studying our GC model described in Section 2.2. Typical experiments made on rodents consist in recording the activity of GC in dMEC while the rat, around 20 cm large, is free to move in a circular enclosure of 2 m diameter (Hafting et al., 2005). In order to run our experiments in almost similar conditions, our robot, around 40 cm large, randomly moves in an hexagonal arena of 4 m diameter (see Figure 5B). Position and simulated GC are simultaneously recorded during all experiments. A basic sensory-motor loop is used to perform obstacles avoidance so that the robot stays inside the hexagonal playground.

The Experiments (6, 7) study the place cells responses in a simulated environment. In a last set of Experiments (8–11), the real environment is composed of two rooms connected by a corridor (see Figure 5C). Different parts of our laboratory are visually similar so that our vision-only based recognition system is subjected to perceptual ambiguities. This is one of the typical problems encountered while the robot tries to visually localize itself in a cue-redundant environment. For analysis purpose, we forced the robot to learn 19 regularly-spaced places (each 1.5 m) on a multi-room path. The learned path (named *path*1) starts in one room, passes through a corridor and ends in a second room very similar to the first one.

#### 2.5.3. Study of the impact of path integration recalibration mechanism over grid cells activity pattern

This section describes Experiments (1–5) conducted to study our model of GC described in Section 2.2.

##### 2.5.3.1. Experiment 1: random exploration without recalibration

In this first experiment, the robot exploration behavior is only based on random movements and the calibration mechanism is not used. This test simply intends to replicate previous observed effect of PI cumulative errors on GC firing pattern. It underlines the need for a mechanism based on other modalities to correct or to limit those errors and maintain stable GC firing pattern.

**Recalibration of path integration via a periodic homing behavior**. It is well-known that reliable visual landmarks in the environment allow to update and correct for errors that occur during PI process (Etienne, 1992; Gallistel, 1993). In these experiments, the recognition of a given place in the arena is used as a specific landmark (identity and azimuth couples). This mechanism provides a PI update. At first, a conditional learning mechanism allows associating a specific place cell with the concomitant activity in the PI field. Next, the activation of the same place cell induces an activity on the PI field corresponding to the activity present during learning. This activity will mash up the ongoing path integration activity and can be considered as a simple recalibration procedure.

**Figure 6:**
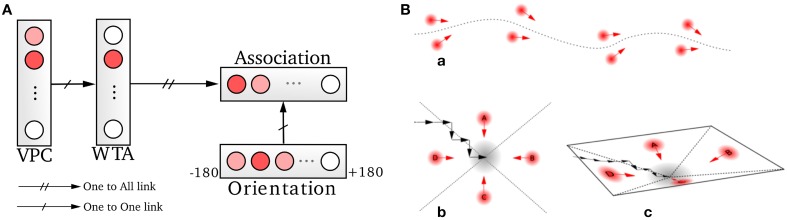
**Homing mechanism based on a simple sensory-motor loop relying on vision. (A)** A particular action (a direction in our case) is associated with each winning visual place cell (VPC). This association is learned by a least mean square algorithm. The system is then able to move in the learned direction when the associated place cell wins the recognition competition. **(B)** This simple mechanism allows the system to exhibit robust behaviors by the use of only few sensory-motor associations (path following, homing task). An attraction bassin emerges from the sensory-motor dynamic.

A homing behavior is used to force the robot to return to the recalibration location. The robotic architecture for this robust homing behavior is based on sensory-motor associations relying on vision (Figure 6) (Gaussier and Zrehen, 1995; Gaussier et al., 2002; Giovannangeli et al., 2006). A visual attraction field is learned around the homing location, allowing the robot to converge autonomously into the resetting area. A simple periodic drive triggers the homing behavior and thus allows the robot to go to a recalibration place by itself every minute. The drive is reset each time the goal is reached, allowing the robot to switch back to a random exploration strategy ^2^.

##### 2.5.3.2. Experiment 2: impact of the recalibration rate

In this experiment, we test the impact of the time elapsed between successive recalibrations over the GC firing pattern. The robot explores the arena for 30 min. Robot linear speed is 20 cm/s and the arena is 4 m large. Hence, the robot needs 20 s to cross the arena from side to side. This implies that the period between two recalibrations must be longer than 20 s to obtain data from everywhere in the environment. Conversely, previous experiments showed that the firing pattern of our GC was scrambled if the period between two recalibrations was longer than 5 min. Thus, we tested the following recalibration timing : 30 s, 1 min, 2 min, and 4 min.

##### 2.5.3.3. Experiment 3: impact of the recalibration location

We study here the impact of this recalibration place inside the enclosure by comparing the results obtained for two recalibration locations. Since the response field of the VPC is quite large as compared to the arena size, another information was used to reduce the reset zone. We chose to use a colored piece of paper (format A4) that could only be detected by a color sensor located below the robot (between its two wheels). This piece of paper is similar to some reward the animal can receive when arriving at some goal location. Similarly to animal experiments, the robot reward (i.e., the red piece of paper) is chosen so that the robot cannot use it to control its motions. The robot camera cannot see the floor near the robot and when the robot is far away the piece of paper, it is too small on the floor to be perceived by its low-resolution vision system. Each experiment runs for 30 min. Every minute, the homing behavior is triggered. The red paper stuck on the floor represents the virtual goal location. In the first Experiment (3a), the tests were performed with a goal (recalibration area) at the center of the arena. In the second Experiment (3b), nothing is changed excepted that the red piece of paper (recalibration area) is set in a corner defined by the two walls and the attraction field is learned to converge to this corner. Note that in this experiment, the ultrasound sensor profile is learned and used to restrict and to better define the recalibration zone.

##### 2.5.3.4. Experiment 4: random exploration

Like in the previous experiment, in the first 30 min of this experiment the robot explores the arena and a homing behavior is triggered every minute to recalibrate PI. But after these first 30 min, visual information and homing behavior are disabled and the robot randomly moves for another 30 min. GC firing pattern is measured after 33, 36, 39, 42, 45, 48, 51, 54, 57 min, and finally 1 h. This experiment differs from Experiment 1, since the robot randomly moves only after a complete exploration of the environment and the establishment of a stable GC pattern.

##### 2.5.3.5. Experiment 5: calibration without vision

In this experiment, the robot explores first entirely the arena for 30 min. During this first phase, it can recalibrate its PI using the homing behavior as in the previous experiment but the activity of the used to define the recalibration location is the result of a simple conditioning rule between visual information, information, ultrasound sensor profile and GCs activities. Next, the robot runs again for 30 min in the same arena, but the visual inputs are disabled and the recalibration of the place cell activity is only driven by GC activities and the recognition of the learned ultrasound profile.

#### 2.5.4. Analysis of the place cells responses

This section describes experiments conducted to study the model described in Section 2.4. In a first set of experiments, place cells result from the same simple WTA learning of GC that in Gaussier et al. (2007) and the impact of several parameters on the GCs responses are studied in simulation. In a second set of experiments, place cell responses of the model showed in Figure 3 are studied using a real robot evolving in an environment composed of two rooms connected by a corridor (part of our laboratory). The activities of the different place cell populations (VPC, PredVPC, MPC) are recorded while the robot is passively moved in this environment and after a kidnapping test (i.e., moving the robot to a previously learned distant place). Note that in all this second set of robot localization experiments, PredVPC neurons rely on GC with analog response (see Section 2.4). Note also that each VPC enough activated can trigger a PI recalibration (i.e., each VPC is associated with a PI value).

**From grid cells to place cells**. Here, we study how GCs can induce place cells relying only on PI. These simulations are performed in a square environment (500 × 500 states).

##### 2.5.4.1. Experiment 6: experiment on learning a place cell from 3 different grid resolutions

Following our previous approach, a simple WTA learning is used to learn GC activity profile and to give rise to like activity. One can refer to Gaussier et al. (2007) for details and equations.

##### 2.5.4.2. Experiment 7: experiment on the effect of parameter values over the generated place field

Here, the generalization performances of these cells are studied. As previously, the same competitive learning mechanism is used. Our studies show that at least two parameters can impact the size of the place fields: the discretization factor and the number of simulated GC. These experiments show the effect of different parameter values on the generated place fields.

**Place cell responses**. These tests underline the behavior of our different populations of place cells in a visually ambiguous environment.

##### 2.5.4.3. Experiment 8: experiment on place cell responses (vpc, predvpc, mpc) over a 23 meters long path

In this experiment, the recognition level of one MPC and the corresponding PredVPC and VPC are recorded while the robot moves passively over a 23 m long path (*path*1).

##### 2.5.4.4. Experiment 9: experiment on the generalization properties over a path

In order to test the robustness and the generalization capabilities of our MPC, a navigation experiment on a 25 × 15 m environment has been performed (see Figure 5C). The learned path (*path*1) starts in one room, passes through a corridor and ends in a second room very similar to the first one. Next, the robot followed 5 different paths parallel to the learned path, passively moved by a remote control. Those five paths allow to cover a large space near the learned path, in order to test the generalizations properties of the model (multimodal place field size). To show the deterministic nature of the results, we repeated the experiment a dozen of times in a changing environment (ambient light and furniture changing, persons moving).

**Solving the Kidnapping problem**. Previously, place cell activities were analyzed along a continuous path in the environment. In the following study how our simple model of MPC behaves when a conflict occurs between both inputs. Several kidnapping events were performed: the robot was transported (lifted and blindfolded) from different position along *path*1 to an other location near this same path. When the robot is at the chosen location, the different inputs are enabled again and the robot is remote-controlled to perform a path along (or near) *path*1. *path*1 is learned before VPCs, PredVPCs, and MPCs activities are recorded during the kidnapping experiments.

##### 2.5.4.5. Experiment 10: robot kidnapped to another room

The robot is remote-controlled and learns to localize itself along *path*1. Next, it is kidnapped (blindfolded and lifted) and moved to another place along the *path*1 (in *room*2). Then, the robot tries to localize itself while being remote-controlled to perform the end of *path*1 again.

##### 2.5.4.6. Experiment 11: robot kidnapped near the beginning of the path

The robot learns again to localize itself along *path*1. When the robot reaches the final position in room 2, it is kidnapped and moved again to its initial position in the first room. It then tries to localize itself while being remote-controlled to perform a path near the learned path.

## 3. Results

### 3.1. Results relying on the impact of path integration calibration on Grid cells activity patterns

#### 3.1.1. Results of experiment 1

**Figure 7:**
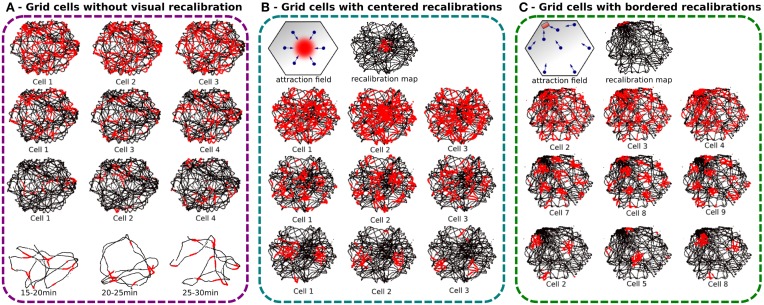
**Experiments made with a real robot randomly moving in an hexagonal enclosure during 30 min**. Robot paths are shown in black, with superimposed single grid cell activity in red. **(A)** Grid cell activity obtained with our neural model without any recalibration (Experiment 1). Top, result of 30 min of navigation without recalibration for 3 different modulo [projection of 60 neurons on, respectively 4 (top), 9 (middle), and 25 neurons (bottom)]. We show 3 randomly chosen cells for each modulo. Activity fields are blurred due to path integration errors. Bottom, 5 min slices of time allow to show periodic firing field. **(B)** Same experiment but with a periodic calibration in the center every minute (Experiment 3a). The origin of the path integration is set in the center of the environment. Top, an attraction field is learned around the center allowing the robot to autonomously converge in the calibration area every minute. Left, 6 different visual place cells are learned to generate a correct attraction field. Right, the recalibration map shows a relatively large resetting area. Bottom, results present more coherent regular patterns than without calibration but the precision can not be smaller than the calibration area. **(C)** Same experiment but with a periodic calibration in a corner of the arena every minute (Experiment 3b). A thinner resetting area is obtained with bordered recalibrations because of the physical edges of the environment that give more precision. Regular hexagonal patterns of different spacing, orientations, and phases are shown.

As expected, cumulative errors on PI quickly degrade regular pattern activities by spreading over neighboring areas so much that the global activity appears randomly distributed over the room like in Gaussier et al. (2007) (see Figure 7A). Each cell presents a blurred activity with no visible tessellation because of the 30 min error accumulation. PI errors come from the discretization of the field, the precision of the internal compass (HD cells) and errors over the displacement length. However, neurons of the GCs network present periodic and more coherent activity when 5 min slices of experiment are considered. As indicated before, a mechanism based on other modalities is needed to limit or correct this drift. In order to solve this problem we decided to associate the PI pattern with VPC activity.

#### 3.1.2. Results of experiment 2

**Figure 8:**
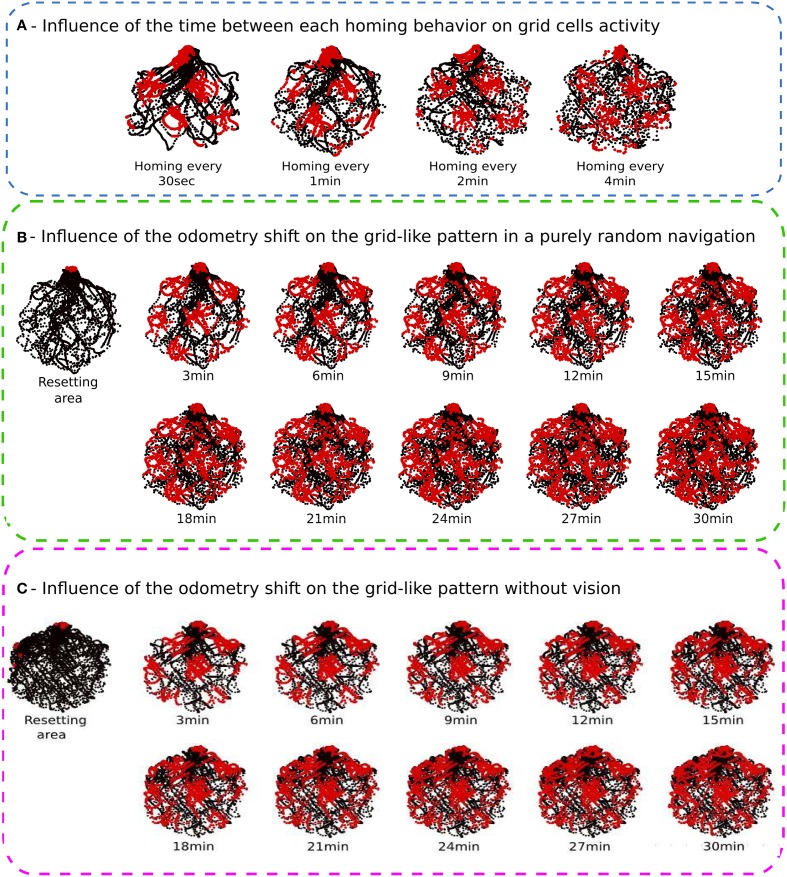
**(A)** Influence of the time between each homing behavior on grid cell activities (Experiment 2). We made 4 Experiments on a real robot randomly moving during 30 min in the hexagonal enclosure. Activity of a grid cell (red dot) superimposed on a robot path (black dot) for different homing periods. From left to right, the robot go recalibrate itself every 30 s, 1 min, 2 min, and 4 min. The shorter the homing period is, the better the pattern activity is defined. But the robot needs a least 1 min to uniformly cover the entire environment. **(B)** Influence of the PI shift on the grid-like pattern in a purely random navigation of 30 min (Experiment 4). As for previous experiments, the robot starts a random navigation in the enclosure and uses sensory-motor associations to go recalibrate itself every minute in a corner. After 30 min, we inhibit the homing strategy and the system continues moving for another 30 min without any calibration. Results show, respectively the resetting area (for the first 30 min), and the activity of a grid cell every 3 min after inhibiting calibration. Blurring activity appears as a gaussian noise with time. **(C)** Influence of the PI shift on the grid-like pattern without vision (Experiment 5). After 30 min of random navigation with a calibration every minute, the robot is blindfolded. It keeps moving for another 30 min without vision. It keeps trying to go back home every minute but only with PI and ultrasound sensors profile. A calibration of PI occurs only when the right place cell, computed from grid cells, is winning and when head-on proximity sensors (US) rise a high-level threshold. Left, resetting area for the entire experiment duration. Reset activity appears distributed in space on the border of the environment. Right, activity of a grid cell every 3 min after blindfolding. Spreading activity appears to be the result of a rotation of the initial grid pattern. Because of inaccurate border calibrations, grid cell activity shifts in orientation but keeps its original spatial periodicity.

The recalibration rate depends on the precision of the PI system and the size of the environment (see Figure 8A). The results show that if the recalibration is performed every 30 s, the grid pattern is sharp but the robot cannot explore uniformly the environment. It stays close to the calibration area. On the contrary, if the calibration is triggered every 4 min, the environment is well-covered, but the activity of the grids starts to be blurred. For the next experiments, the calibration rate is set to 1 min since this duration is a good compromise ratio between precision and the surface that can be covered by the robot.

#### 3.1.3. Results of experiment 3

The results presented on Figure 7 — demonstrate that the precision of the generated grids highly depends on the size of the recalibration area which itself depends on the VPC properties and on the competition mechanism to select the winning cell. Location of this recalibration area can really matter. If a VPC has been learned near the center of the environment, its place field can be really larger than if it has been learned near a border of the environment. As a matter of fact, at the center of the environment (far away from the landmarks), one elementary displacement generates smaller azimuth variations than near the border of the environment where the landmarks are usually located. Figures 7B,C indicate that the winning zone of a VPC is more precise if it has been learned near a border.

In the first experiment, with recalibrations in the center of the arena, the results show an error field relatively large since it represents almost 20% of the arena width.

The second experiment tries to solve the precision problem by using walls of the arena to define a smaller recalibration area. Indeed, the corner of 2 walls represents a natural singularity where it is easy to converge. This technique uses the conjunction of ultrasound sensors activity profile and place cells recognition to increase precision. Ultrasound sensors activity profile is learned and categorized at the recalibration location and helps to reduce errors introduced by vision-only based recalibration. During the homing behavior, the reset area is then more precisely defined in the corner of a room since the edges of the enclosure help to define a precise area thanks to the dynamical properties of the obstacle avoidance mechanism used and thanks to the help of the proximity sensors bringing new data to discriminate between places near the borders and the other places. The results present well-defined pattern of GC for a recalibration point near a corner. As before, the different grids share the same spacing when using the same modulo and the same orientation for a given discretization factor but the results are here more visible. We can clearly see on Figure 7C that each cell produces a grid activity with a specific phase.

#### 3.1.4. Results of experiment 4

Without recalibration, results show a degradation of the grid pattern over 30 min of random movements (see Figure 8B). Blurred activity appears progressively as a gaussian noise depending of the elapsed time. The activity pattern of the GCs seems totally random after 15 min.

#### 3.1.5. Results of experiment 5

Results presented in Figure 8C highlight the important role of vision in maintaining precise and coherent grid-like activities since in the absence of any external cues, the GCs loose progressively their angular precision. The robot still tries to recalibrate by using the available information (PI and ultrasound activity profile) but the fast drift of the PI results in a mismatch in the corner used for calibration. It results in a 60° rotation of the grid pattern around the center of the arena. Because of inaccurate border calibrations, GC activity shifts in orientation but keeps its original spatial periodicity. Moreover, after 20 min of experiment, the cumulative errors in distance integration become so high that the calibration place is perceived outside the arena. Consequently, the robot cannot recalibrate for the last 10 min of experiment.

### 3.2. Results relying on place cells from grid cells

#### 3.2.1. Results of experiment 6

The activity of a place cell built from the learning of a binary GC pattern exhibits a very narrow place field (see Figure 4A). Note that such a place field is a 3-stepped stair because of the three different grid resolutions (3 modulo).

#### 3.2.2. Results of experiment 7

Results obtained with a large number of GC indicate that the discretization of the PI field (number of neurons of field *E^i^* used to code the value of *D_i_*) has a direct impact on the place field width (see Figure 9.1). The number of GC used to generate place cells affects the field resolution (see Figure 9.2). The more GC the system uses, the better the resolution of the place field is.

**Figure 9:**
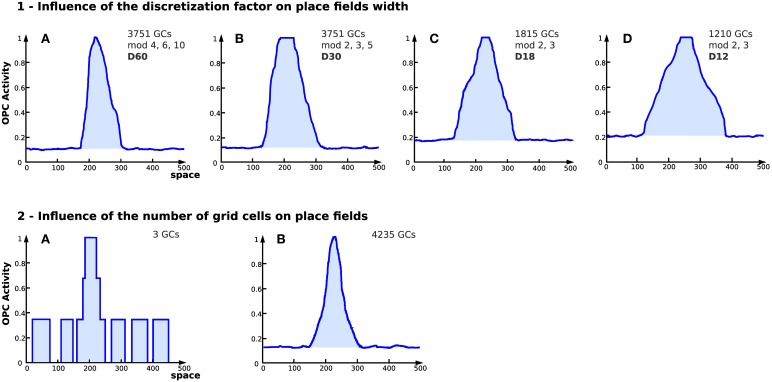
**Influence of discretization and number of grid cells on place fields in simulation (Experiment 7)**. On each figure, the x axis represents space (the distance from the starting point in an abstract unit) while the y axis represents the activity of a place cell (a WTA learns the conjunction of grid cells, see Section 2.4). **(1)** Influence of the discretization factor on place fields width. Place cell obtained from grid cells depending on different parameters [4 different setups (*GCs*) corresponds to the number of grid cells used, *mod* refers to the different modulo and *D* refers to the discretization factor]. The discretization factor has a direct impact on the field width. Note that the smaller the discretization factor, the higher the noise. **(2)** Place cell field obtained from grid cells with different modulo and 2 different numbers of cells for the same discretization factor (*D* = 60). (A) 3 grid cells. (B) 4235 grid cells. Increasing the number of GCs tends to reduce the noise level and enhances the resolution of the generated place field.

#### 3.2.3. Results of experiment 8

**Figure 10:**
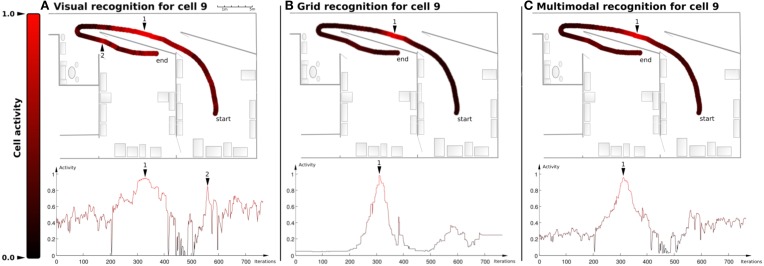
**Example of recognition for one cell (Experiment 8)**. On the upper part: Activity plotted on one path (red = 1, black = 0). On the lower part: Same activity plotted in time. **(A)** Visual place cell 9 shows maximum activity on the learned place (1) but also on place (2) because of cue redundancies. **(B)** Odometric place cell 9 (PredVPC) exhibits a well-defined shape at the learned position (1) and no ambiguity. **(C)** Merging the 2 allows the system to disambiguate vision. Results present a correct activity for the multimodal place cell (MPC) 9.

Figure 10 presents the recognition activity for different PC populations (VPC, PredVPC, MPC) while the robot is moving between two large rooms in our laboratory. The recognition level for one VPC shows high activity in two different places because of cue redundancies (see Figure 10A). Activity of the corresponding PredVPC cell exhibits a well-defined shape at the learned position (see Figure 10B). The merging of PredVPC and VPC defines Multimodal Place Cells (MPC) allowing the system to solve the visual ambiguities of the VPC (see Figure 10C).

#### 3.2.4. Results of experiment 9

**Figure 11:**
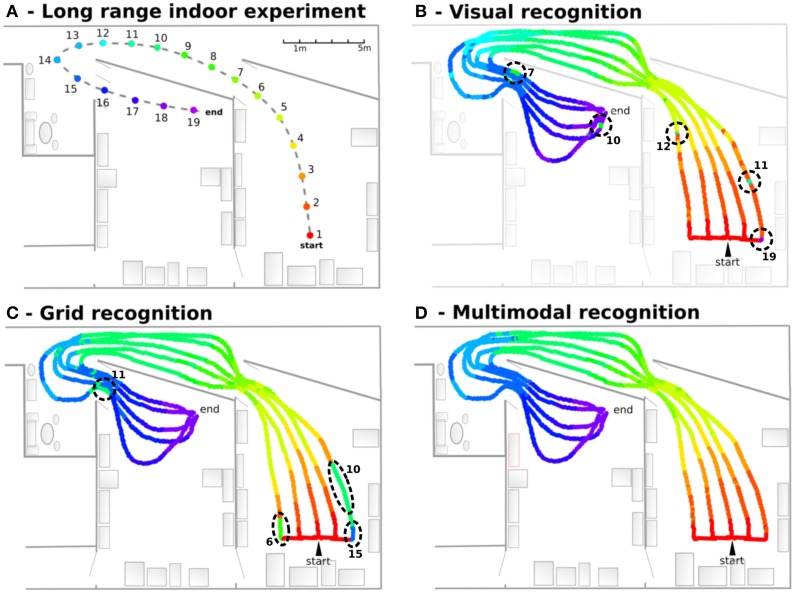
**Navigation in an indoor environment (Experiment 9)**. **(A)** Experimental setup: The robot learns 19 regularly-spaced places (each 1.5 m), starting from a room, passing through a corridor and ending on a second room. **(B)** Visual recognition obtained for 5 different paths. Each color is associated to one visual place cell. Results show great generalization capabilities but present ambiguities (dotted circles. Numbers correspond to indexes of perceived places.). **(C)** Grid recognition for the same paths. Grid fields are smaller but without any ambiguities. A place is not recognized if the robot is too far away from the learned place. **(D)** Multimodal recognition obtained by merging visual and grid place cells. The synergy of both modalities shows well-defined areas even if the robot is far from the learned path.

In this experiment, the robot learns 19 regularly-spaced positions (each 1.5 m), starting from one room, passing through a corridor and ending in a second room. VPCs, PredVPCs, and MPCs activities are recorded for 5 different paths (see Figure 11). Recognition of places from visual information alone shows great generalization capabilities but presents ambiguities. Grid recognition for the same paths leads to smaller fields than visual place fields but does not suffer any ambiguity. However, a place cannot be recognized correctly with the PC computed from the GC if the robot is too far away from the recalibration area (because of the cumulative PI error). Multimodal recognition obtained by merging VPCs and PredVPCs demonstrates the benefit of both modalities. This lead to well-defined PC even if the robot is far from the learned path.

#### 3.2.5. Results of experiment 10

This experiment (see Figure 12.1) showed that the VPC quickly localize the robot after the kidnapping event when the grid recognition stays totally wrong until a PI recalibration occurs. Every time the activity of the winning VPC is not well-defined, the robot must run typically 5 meters before being able to recalibrate its PI. Nevertheless, MPC exhibit coherent results even if the recalibration signal does not yet appear.

**Figure 12:**
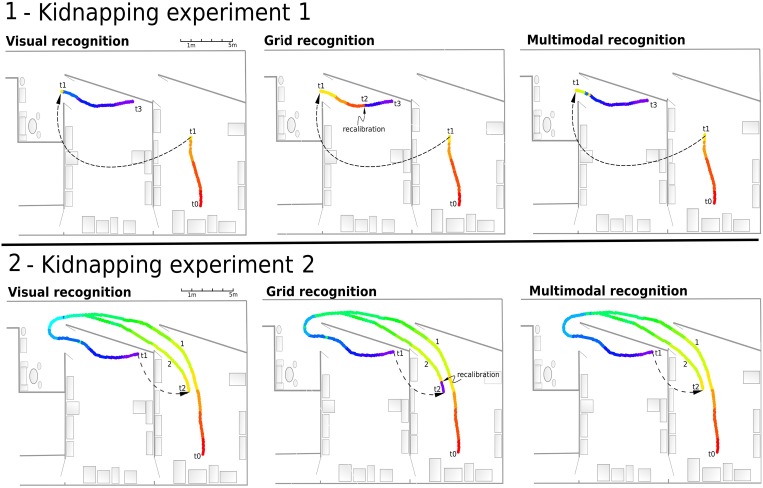
**(1)** Experiment 10. Solving the kidnapping problem: The robot is remote-controlled from the starting point (t0) in a first room to (t1) in our laboratory. Next, it is kidnapped (blindfolded and lifted) and moved to an other room (t2). Then, the robot tries to localize itself while it is remote-controlled to the end (t1–t4). Left - Visual recognition during the experiment. The visual recognition system instantly localizes the robot after the kidnapping event. Middle - Grid recognition during the same experiment. Once kidnapped, grid recognition stays totally wrong since there is no recalibration signal coming from vision. The signal coming from vision (t2) allows to recalibrate the path integration field to retrieve a coherent grid recognition. Right - Multimodal recognition shows coherent results even if recalibration signal does not yet appear. **(2)** Experiment 11. Solving another kidnapping problem: The robot passively moves from t0 to t1 following the first path (1). It is then kidnapped and moved to the initial room (t2). It then tries to localize itself while being remote-controlled on the second path (2).

#### 3.2.6. Results of experiment 11

In this experiment (see Figure 12.2), the MPC are able again to quickly recognize the correct location thanks to the visual information. Recalibration of the PI can occur earlier because of the good generalization properties of the previously learned VPC. When the robot is in an already known and highly recognized place, it recalibrates its PI field to a previously learned value. As previously mentioned, this recalibration mechanism allows the system to keep consistency between VPC and PredVPC and prevents from being lost near learned locations. Moreover, thanks to the merging mechanism, the perceived location does not stay wrong after a displacement of more than 2 m. Indeed, even if the PredVPC activity decreases progressively while getting far from learned places, the VPCs do not suffer from drift problems and can delete the errors induced by the GC system.

## 4. Discussion

### 4.1. Grid cell model

Following our previous works on bio-inspired robot architecture for navigation (Cuperlier et al., 2007; Gaussier et al., 2007), we proposed in this paper a model of GC that does not require prior buildup of a Cartesian map of the environment. Contrary to other models based on attractor dynamics or models based on interferences, this model does not require complex neuronal properties for the entorhinal cortex (no need of a particular neuron dynamics as in Fuhs and Touretzky, 2006; McNaughton et al., 2006). Moreover, our model differs from other works by several other points listed above.

#### 4.1.1. Grid cells from modulo projection of path integration

**Figure 13:**
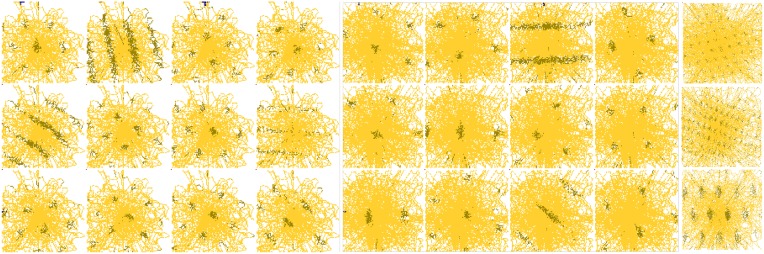
**Variety of grid cell activity obtained in a simulated environment for different parameters**. Grid cells are computed by random modulo projections. Activity of 27 cells randomly picked from a population of 30,000 cells. The simulated robot explored the square environment for 20,000 iterations. Resulting patterns exhibit all kind of activities (including grid-like with different spatial periods, phases, gridness, and orientations).

First, the GC pattern observed is only resulting from the projection and merging (product) on the dMEC neurons of PI PI information. These projections simply act as various modulo's operators applied on path integration involve complex operations. To verify whether a strict modulo operator is necessary to obtain GC activities, we performed simulations of EC neurons that were learned autonomously by a network connecting the dMEC granular cells to a limited and random number of connections with the neurons associated to the PI field. The results show that a wide variety of activity patterns, including grid-like patterns, can be obtained by relaxing the constraints on the model (prime constraints over the modulo operator or imperfect modulo projection: see Figure 13 and Gaussier et al., 2007).

From a neurobiological point of view, it seems that EC is not the main place where PI is computed, but is instead a place where a generic code is formed to allow better recognition or prediction. More precisely, we argue that the neurons in EC provide a code that compresses other cortical information so that the hippocampus would not need to perform specific computation for place recognition but would use generic neuron properties (scalar product and competition) to learn and recognize complex cortical states involving several cortical areas. For instance, the same modulo operation could be performed both on the azimuths of visual landmarks and on the neurons coding the recognition (identity) of visual landmarks. A product of these signals can then be learned by the hippocampus. A recent work (Killian et al., 2012) has shown that GC patterns could also be observed in primate by a simple visual scene exploration without any locomotion. Moreover, even if in this paper our work was focused on a spatial task, we can hypothesize that other cortical inputs could also benefit from the same process (like odors or sounds). Thus, our model predicts that code based on the product of several modulo projections is a general property of EC that is not specific to spatial information. Activity of a given neuron of EC (X(t)), receiving *i* different signals from *n* different modalities, can be given by:

(13) $$X(t)=\Pi (Signal_{i}(t)\;mod\;scale_{i}(t))\text{with i}\in 1,\;2,\ldots n.$$

The different patterns activating a given neuron should be uncorrelated both temporally and spatially to minimize ambiguity.

#### 4.1.2. A grid cell model based on extra dMEC path integration

Second, our model contrasts with most of the other GC models as it does not assume PI is performed by GC themselves from HD cells information or velocity information. Instead, we make the assumption that long range PI could be stored in other brain areas. At least two brain regions could generate such an extra hippocampal PI: the retrosplenial cortex (Cho and Sharp, 2001) and the parietal cortex (Etienne and Jeffery, 2004; Parron and Save, 2004).

Note also that the recalibration of the PI can be processed outside the entorhinal cortex. The retrosplenial cortex may be that place since several works show that this area is involved in the association process between visuospatial and idiothetic information, namely *“it places visual context information within a framework of idiothetic knowledge”* (Cooper and Mizumori, 1999; Mizumori et al., 2001).

#### 4.1.3. Getting stable grid cell patterns from robotic experiments

Experiments (1–5) performed on the recalibration mechanism can give several clues about how to setup correctly the model to perform other sensory-motor navigation tasks (like place-action sequences). Note that even if calibration is needed to maintain a correct grid pattern over a long period, GC patterns appear complete in the robot first visit of an environment. Indeed, our simulated GC present periodic and coherent activity when we only consider 5 min slices of experiment (see results in Section 3.1).

Moreover, we addressed the question of the calibration frequency and the choice of the recalibration point. Experimental results of Section 3.1 indicate that our robot must recalibrate after running at most 48 m. Hence in a large environment, the recalibration has not to be too frequent. Indeed, recalibration can occur when a place of the sequence is well-enough recognized. This leads to the definition of a level of confidence in the visual place cells recognition. Experiment 3 on the location of the goal underline the same key point, since GC activities are better defined if the calibration zone is small.

One could notice the qualitative nature of our results. Indeed, trying to perform many experiments on a real robot is quite long and difficult if one consider all technical aspects such as robot velocity, battery life-time, dynamic environment, and complexity of the hardware architecture. In this article, we decided to focus on robotic experiments that illustrate our model. Future work will focus on quantifying the quality of the present model by providing precise statistical analyses for the different experiments presented in this article.

#### 4.1.4. About path integration calibration sources

As expected, errors in PI accumulate due to slipping wheels and discretization errors. This prevents correct GC activities after 4 or 5 min of exploration. Hence, other information have to be taken into account to correct these errors. Therefore, we chose to exploit visual information via a simple conditioning mechanism linking VPC and GC to limit PI error. One other possibility could be the use of border cells that have been discovered by Solstad et al. (2008). Those cells are mainly activated when the animal reaches a limit of its environment like a wall and they seem to be related to the tactile sense (a transparent wall also activates them). An interesting solution could be provided by a recent work that presents an attempt to take into account border cells activities in order to reduce the PI drift (Cheung et al., 2012).

#### 4.1.5. How does this model take into account biological results

**Figure 14:**
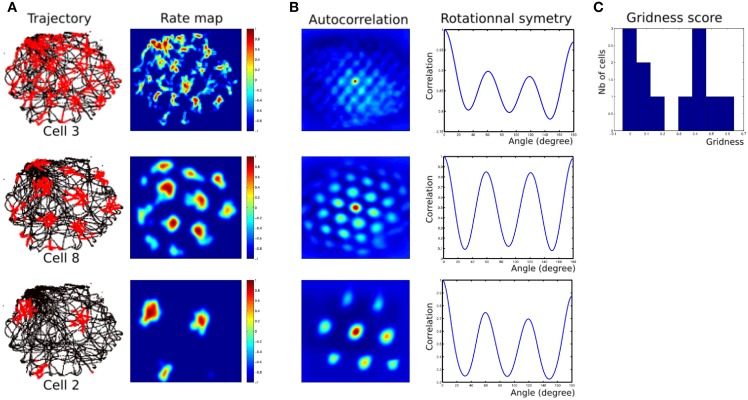
**(A)** Firing field of grid cells with 3 different spatial frequencies (one cell from each layer). Left row, robot path (black) with superimposed grid cell activity (red); right row, rate-coding map (obtained by a gaussian convolution on cell activity). Red = 1, dark-blue = 0. **(B)** Periodic structure of grid cells activity shown in panel **(A)**. Left row, autocorrelation matrix for the rate map. The color scale is from blue = −1 (anticorrelated) through green = 0 (no correlation) to red = 1 (fully correlated). Distance scales are 4 times bigger than for the rate map. Right row, angular periodicity of the autocorrelation matrix. We rotated the autocorrelation map from 0 to 180° (step of 1°) and computed the pearson correlation between each rotated matrix and the original one. Grid structure appears as a sinusoidal modulation of this correlation, with peaks at multiple of 60°. Correlations are 1 at 180° because of the mirror symmetry. **(C)** Histogram of gridness score for 12 cells recorded in all 3 layers of our MEC model.

Robotic Experiments (2, 3) show that we can find parameters (homing frequency and goal location) allowing taking into account some results found in experiments made on rats. Namely, results of Figure 14 present rotational symmetry and auto-correlation values quite close to those found on animals (see Sargolini et al., 2006). Parameters of the model can control orientations, spacing and phases of the generated grids.

It has been shown that when visual cues are present, they exert a strong control over the alignment of grids: rotating a cue card on the wall of a cylinder causes grid patterns to rotate by the same amount (Hafting et al., 2005). Our model can not directly replicate these findings. This is mainly due to the use of a magnetic compass device in input of our HD cell, but we strongly suggest that replacing this magnetic device by a visual one should allow to provide similar results (Giovannangeli and Gaussier, 2007; Delarboulas et al., 2014). More experiments have to be done on this.

Contrary to the robotic model proposed in Milford et al. (2010), we do not need to take into account recent findings about the conjunctive coding of position, orientation and velocity as reported in Sargolini et al. (2006). First, conjunctive and non-conjunctive GC do not seem to be located in the same area of EC and are not present in the same proportions. Second, we presented in Gaussier et al. (2007) a more complete model describing a loop between hippocampus, entorhinal cortex and the subiculum area that could explain conjunctive activity exhibited by dMEC neurons. More work has to be done to discuss in details these data but the architecture we proposed in this paper seems very appropriate to perform such study.

### 4.2. From grid cells to place cell

#### 4.2.1. How parameters of our grid cell model can impact place cells

This work expands previous work on GC (Gaussier et al., 2007) showing how GC patterns can be processed by a competitive learning mechanism (WTA) to give rise to place cell activity. The size of the resulting place fields can be modulated by the discretization factor of the place integration field and the number of GC used to generate such place cells. The place field size is an important parameter for place-action navigation since it defines how far from the exact learned position the associated movement will be generated, namely the generalization property of the place cell/action mechanism.

#### 4.2.2. Multimodal place cell model

As previously explained, all grids present binary fields (activated or not) so that the pattern generated by the conjunction of three grids is a three-steps stair shape. Narrow place cells are treated as if there were no proximity distance between them. From the study of the parameters for our GC model, we learned that one could get larger place fields but it requires a very large number of GC to get a correct resolution. We can argue that biological systems benefit from a large number of GC with different characteristics allowing to generate a natural continuity and these large place fields. Simulations using a large number of GC advocate our claim (see Figures 9.1,9.2). Nevertheless, considering the computational aspects, we chose to keep only 3 different grid networks (generating our three-steps stair shaped activity) and to generate analog activity by adding lateral connections between the grid related GC (GC coding for the same spacing). This technique allows spreading activities field over neighboring cells by using a torus topology (McNaughton et al., 2006), and so generates continuity (see Figure 4). Yet, in this case, our model looses the ability to distinguish between two near places if the distance between them is smaller than the mask size.

Even, if a simple competitive learning of GC is able to give rise to place cell activity it is not enough to explain hippocampal place cell activity. Indeed, these place cells are also driven by visual input and input of other modalities (odor, tactile…) and they do not seem to be of equal importance (Maaswinkel and Whishaw, 1999). We thus presented a new robotic architecture for the emergence of place cells from both vMEC (VPC) and dMEC (PredVPC) inputs. In this model a simple associative learning allows GC activities to predict VPC and to merge both modalities in a common representation. Note that in the proposed model GC responses are associated with VPC but a reverse association is also plausible (prediction of the grid patterns from VPC). In our model, PI recalibration is based on such an association (even if applied directly to PI and not to GC).

#### 4.2.3. Solving a visual ambiguity problem

As mentioned in introduction, merging allothetic, and idiothetic information is not new in robotics, for instance in Martinelli et al. (2007) the authors present a model relying on an EKF (McElhoe, 1966) to merge the observation coming from a laser range sensor with the robot odometry. Numerous works based on other methods (like particle filter) can also be found in Thrun (2003) and Thrun et al. (2005). Similar fusion are also be described in several bio-inspired robot architectures (Filliat, 2003). However, little is known about how exactly such fusion is processed in animal brain and what is the role played by each input according to the navigational behavior of the animal. Thus, we also provide in this paper a very simple model showing that our GC model can also be used as an input to perform and to study such fusion. Like in other studies, the resulting multimodal place code is more robust than place code based on vision only as it reduces visual ambiguities. Indeed, in Experiments (8–11), the two rooms of our laboratory shared a lot of similarities so that our visual-only based recognition system was subject to perceptual ambiguities. This is the major problem we encountered while the robot tried to visually localize itself in a cue-redundant environment. It is not particularly annoying for neighbor places whose cells share a similar sensori-motor behavior (i.e., same associated action and same proposed movement direction). Nevertheless, it can be more dramatic when an ambiguity appears between two close place cells that should be associated with opposite movement directions (for instance, when the robot learns a path with sharp curvatures).

Hence, results show that the visual recognition system allows a great generalization capability (large place field) but presents small perception mistakes due to cue redundancy. Indeed some VPC fire in 2 or 3 different places in the environment.

In contrary, PredVPCs computed from GC present well-defined place fields without any ambiguity. But they are subject to the cumulative errors of PI. This results in a very precise discrimination for small scales but a shifted localization for large scales.

Because of the complementary nature of those two modalities (absolute vs. relative), they present supplementary characteristics that can be used to generate robust localization. As expected, results point out that merging two modalities reduces uncorrelated activities and thus allows getting correct localization without any ambiguity. Finally, MPC are robust and keep large generalization properties ^3^.

#### 4.2.4. Solving the kidnapping problem

Kidnapping Experiments (10, 11) results underline how the different elements of our recognition system behave when the robot is suddenly teleported to a different place. This kidnapping allows putting in conflict inputs of the MPC. By design, kidnapping impacts PI but does not disturb VPC since the visual recognition system quickly localizes the robot after the kidnapping event. MPC can thus benefit from this VPC activity and give a coherent response quickly.

Once kidnapped, grid recognition stays totally wrong until a recalibration signal comes from vision. This signal is triggered when the recognition level of the winning VPC meets several conditions. We chose to trigger the recalibration according to particular thresholds on VPC activity for simplicity. Even if it is not biologically plausible, this mechanism allows getting a correct localization within the first meters following the kidnapping. Moreover, as previously mentioned, other modalities can be taken into account to trigger this recalibration and little is known on these mechanisms.

Future work will investigate how our model behaves when the robot faces radical environmental changes. Strong visual changes can occur in real dynamical environments when for example people move, objects are displaced or when doors are opened or closed. How can a Human get a coherent spatial recognition in these conditions ? For instance, what happens when two previously and separately explored environments become linked (a door is opened) ? How is it possible to merge GC location information based on different starting points? Note that these questions are also related to the *closing loop problem* in robotic simultaneous localization and mapping. Based on our current model, we will investigate the effect of the NLMS learning rate parameter over the spatial response of our place cells. We believe such a learning rule should stabilize the learning in a coherent set of associations since if two neighbor place cells propose simultaneously to recalibrate the PI field with two different profiles an averaged profile can be learned (if the profiles are not too far away) or one of them can win (bifurcation behavior related to the dynamical properties of the neural fields Amari, 1977) leading to a filtering and a stabilization of the different coordinate systems.

## 5. Conclusion

In this paper, we have first shown a neural architecture that can take into account experimental results of GC firing properties on a real robot. We demonstrated the need for a calibration mechanism driven by a periodic homing behavior to keep coherent and precise grid-like properties. We then generalized this calibration mechanism not only to the goal location but also to any VPC learned during a path. In previous work (Gaussier et al., 2007), we showed how GC activity could be merged into place cells by a simple competitive learning rule. But resulting place cells exhibited very narrow place fields with no generalization properties. This raises the issue about how such mechanism could be used by animals to navigate in their environment. In this paper, we claim that these generalization properties could emerge from the huge number of existing GC in the different layer of dMEC. Simulation of a large number of GC shows how the discretization factor and the number of GC can control the size of the place fields. An alternative solution would be to use lateral diffusion of activity between GC having the same spacing (in a way similar to the lateral interactions between cortical columns in the visual system for instance). Neurobiological studies involving selective inactivation of lateral connections could perhaps help to decide if one of these hypotheses is incorrect. Finally, we presented a new robotic architecture for the emergence of place cells from both vMEC and dMEC inputs in order to study the behavioral robustness of our GC. Even if this kind of fusion is not new, our work show how a simple merging mechanism based on conditional association of VPC and GC can give rise to robust MPC. Moreover, this model allows studying the activity of place cells coming from both sources of information. Future studies will focus on the effect of the weight of each information source over the robustness of MPC. How these weights could be learned on-line according to an evaluation of the recognition accuracy of each source has also to be addressed.

In conclusion, experiments performed with a real robot have underlined the need for taking into account sensory-motor behaviors to analyze the performances of a GC model. The need of closing the sensory-motor loop implies some important constraints on the model coherence since the different data flows must use the same coding to be merged. The robotics experiments have enlighten the need of GC redundancy to avoid important errors in the place recognition computed from the GC. As a matter of fact, if the GC are binary, a given GC coding for a long distance spacing will have the same impact on the place recognition than a grid associated to a short spacing. This induces that noise on the GC can generate with the same probability small or very large errors in the place estimation since one bit of error (one neuron activated or not) could be associate to a very large or very small grid. Adding redundancy (using a very large population of GC) suppresses this issue since neurons associated to large grids should be more represented in dMEC than neurons associated to small grids. Adding lateral diffusions on the grids relaxes also the constraints on the number of grids necessary to obtain place fields with good generalization properties. The notion of generalization capabilities is usually absent from GC and place cells models since this notion is related, in our case, to behavioral constraints: learning an homing behavior or a route as a sensory-motor attraction basin. Of course, no one can be sure that the animals navigate using such simple competitive sensory-motor strategies. Yet, one can imagine more complex strategies should face harder problems and thus our criterions to build robust GC and place cells should be considered when building new models of GC or place cells. Moreover, our model suggests that the GC effect is related to a projection/compression property of the cortical or sub-cortical inputs arriving onto the entorhinal cortex. This property can apply to all kinds of sensory information (visual, auditory, tactile…) and could be primarily used as a way to allow the hippocampus to code for any cortical activity and to detect/predict the transitions between them (see our model of transition cells in the hippocampus Gaussier et al., 2002). Since the number of neurons in EC (the entorhinal cortex, the main entrance to the hippocampus) is only a fraction of the number of neurons in the cortex (from 1/29 to 1/2500 for rats and humans, respectively^4^). We can estimate that an important compression factor must be used to represent any cortical activity onto EC2 the superficial layers of the EC (which represents less than 1/10 of the EC neurons). This implies a large number of cortical neurons project onto the same EC neurons. To allow the hippocampus to perform novelty detection and fast learning, it is necessary that for the detection of correlated cortical activities, these activities do not project on the same neuron. Since, uncorrelated sources of information cannot be known in advance (their status can change according to the task) one simple solution is to use different random projections using for instance prime modulo for maximizing the efficiency of the coding while allowing simple Hebbian learning rule to be used to learn and detect correlation in the hippocampus. Hence, our model can be seen as a caricature of what could be the projections from the associative cortical areas to EC and the hippocampus. It represents also an interesting way for autonomous robots to allow fast and efficient detection and learning of state transition.

### Conflict of interest statement

The authors declare that the research was conducted in the absence of any commercial or financial relationships that could be construed as a potential conflict of interest.

## Footnotes

^1^In robotics, merging such multimodal information to get a robust spatial response is usually performed with an Extended kalman Filter (McElhoe, 1966) or a particle filter (Thrun et al., 2001).

^2^A video showing a 30 min robotic experiment replicating the Hafting experiment of 2005 is available at this web address: http://www.etis.ensea.fr/~neurocyber/Videos/homing/30min_hafting_expiment.avi

^3^A video demonstrating this architecture performing a path following task at the “Ecole polytechnique de Palaiseau” is available at this address: http://www.etis.ensea.fr/~neurocyber/Videos/homing/Navigation_Polytechnique_Jauffret_2011_v2.avi

^4^If we consider rats have roughly 20,000,000 cortical neurons and 690,000 EC neurons, while in the human brain, we would have around 20,000,000,000 cortical neurons for 8,000,000 EC neurons (Andersen et al., 2006).

## 6. Appendix

### 6.1. Model parameters

Parameters for visual keypoints extraction:

**Table:**

**Parameter**	**Value**
σ_1_	5.0
σ_2_	2.0
Mask size	10
Threshold	5
Competition radius	18

Log-polar transform parameters:

**Table:**

**Parameter**	**Value**
Circular image radius	18

Panorama parameters:

**Table:**

**Parameter**	**Value**
Number of images in a panorama	15
Panorama field of view	360

Parameters for vision neural networks:

**Table:**

**Parameter**	**Value**
Local view size	16 × 16 pixels
Number of landmarks extracted per image	5
Landmarks network size	3000 neurons
PC network size	25 neurons

Parameters for motor control and path integration:

**Table:**

**Parameter**	**Value**
Action layer (Neural field) size	361 neurons
Path Integration (PI) field size	121 neurons
PI Recalibration, absolute threshold	0.88
PI Recalibration, relative threshold	0.38

### 6.2. Experiment parameters

Robotic platform:

**Table:**

**Parameter**	**Value**
Robotic platform	Robulab 10 from Robosoft
Robot width	40 cm
Robot height	1 m
Number of ultrasound sensors	10
Position of ultrasound sensors	Front: 6 ; Back: 4.
Sensors sensitivity	From 1 cm to 40 cm.
Driven wheels	total: 2 ; left: 1 ; right: 1
Free wheels	total: 2 ; front: 1 ; back: 1
Odometry resolution	1 cm.
Robot linear speed	around 20 cm/s
Magnetic compass resolution: ±3°Embedded CPU	Intel I5 (2.40 Ghz).
Camera resolution	320 × 240 pixels.

Grid cells parameters for Experiments (1–5) in the circular enclosure of 2 m:

**Table:**

**Grid parameter used**	**Number of neurons**
Small grid:	2 × 2 = 4 neurons
Middle grid:	3 × 3 = 9 neurons
Big grid:	5 × 5 = 25 neurons
α parameter in PI :	0.02

Grid cells parameters used in simulation Experiments (6–7):

**Table:**

**Grid parameter used**	**Number of neurons**
Modulo 1:	60 × 121 = 7260 neurons
Modulo 2:	10 × 121 = 4840 neurons
Modulo 3:	30 × 121 = 3630 neurons
Modulo 4:	24 × 121 = 2904 neurons
Modulo 5:	20 × 121 = 2420 neurons
Modulo 6:	15 × 121 = 1815 neurons
Modulo 7:	12 × 121 = 1452 neurons

Grid cells parameters for experiments performed in two rooms and a corridor of our laboratory (8–11):

**Table:**

**Grid parameter used**	**Number of neurons**
Small grid:	13 × 13 = 169 neurons
Middle grid:	17 × 17 = 289 neurons
Big grid:	19 × 19 = 361 neurons
α parameter in PI :	0.02

Path integration and recalibration fields:

**Table:**

**Path Integration field (*D*) size :**	**121 neurons**
Recalibration field size :	121 neurons
Elementary movement field (*V*) size :	121 neurons

VPC activity thresholds for path integration recalibration

**Table:**

**Absolute threshold :**	**0.88**
Activity difference between two most activated VPC (relative threshold):	0.38
